# Quetiapine monotherapy in acute phase for major depressive disorder: a meta-analysis of randomized, placebo-controlled trials

**DOI:** 10.1186/1471-244X-12-160

**Published:** 2012-09-27

**Authors:** Narong Maneeton, Benchalak Maneeton, Manit Srisurapanont, Stephen D Martin

**Affiliations:** 1 Department of Psychiatry, Faculty of Medicine, Chiang Mai University, Chiang Mai, Thailand; 2 Brandon Lane Neuropsychiatry Clinic, Durham, United Kingdom

## Abstract

**Background:**

Schizophrenia and bipolar depression trials suggest that quetiapine may have an antidepressant effect.

**Objectives:**

This meta-analysis aimed to determine the efficacy, acceptability and tolerability of quetiapine treatment for major depressive disorder (MDD). Only the randomized controlled trials (RCTs) comparison between quetiapine and placebo were included. The authors searched such clinical trials carried out between 1991 and February 2012.

**Data sources:**

MEDLINE, EMBASE, CINHL, PsycINFO and Cochrane Controlled Trials Register were searched in February 2012. Study populations comprised adults with MDD or major depression.

**Study eligible criteria, participants and interventions:**

Eligible studies were randomized, placebo-controlled trials of quetiapine monotherapy carried out in adults with MDD and presenting endpoint outcomes relevant to: i) depression severity, ii) response rate, iii) overall discontinuation rate, or iv) discontinuation rate due to adverse events. No language restriction was applied.

**Study appraisal and synthesis methods:**

All abstracts identified by the electronic searches were examined. The full reports of relevant studies were assessed, and the data of interest were extracted. Based on the Cochrane methods of bias assessment, risks of bias were determined. The studies with two risks or less were included. The efficacy outcomes were the mean change scores of depression rating scales, the overall response rate, and the overall remission rates. The overall discontinuation rate was considered as a measure of acceptability. The discontinuation rate due to adverse events was a measure of tolerability. Relative risks (RRs) and weighted mean differences (WMDs) with 95% confidence intervals (CIs) were computed by using a random effect model.

**Results:**

A total of 1,497 participants in three RCTs were included. All trials examined the quetiapine extended-release (XR). The pooled mean change scores of the Montgomery-Asberg Depression Rating Scale (MADRS) and the Hamilton Depression Rating Scale (HAM-D) of the quetiapine-treated group were higher than those of the placebo-treated group with the WMDs (95%CI) of -3.37 (-3.95, -2.79) and -2.46 (-3.47, -1.45), respectively. All studies defined the response and remission as ≥ 50% reduction of the MADRS total score and the MADRS total score of ≤8 at endpoint, respectively. The overall response and remission rates were significantly greater in the quetiapine-treated group with RRs (95%CIs) of 1.44 (1.26, 1.64) and 1.37 (1.12, 1.68), respectively. The pooled discontinuation rate was not significantly different between groups with an RR (95%CI) of 1.16 (0.97, 1.39). The pooled discontinuation rate due to adverse event was greater in the quetiapine group with an RR (95%CI) of 2.90 (1.87, 4.48). With respect to sleep time, the pooled mean change Pittsburgh Sleep Quality Index (PSQI) scores of the quetiapine-treated group was also significantly higher than that of the placebo-treated group [WMD (95%CI) of -1.21 (-1.81, -0.61)].

**Limitations:**

Variety of quetiapine XR doses and the small number of RCTs were key limitations of this meta-analysis.

**Conclusions:**

Based on the limited evidence obtained from three RCTs, quetiapine XR is effective for adult patients with MDD. The high dropout rate due to adverse events suggests that some MDD patients may not be able to tolerate quetiapine XR. Due to the balance of its efficacy benefit and risk of side effects, as the overall discontinuation rate shown, the acceptability of this agent is not more than placebo. These results should be viewed as the very preliminary one. Further studies in this area are warranted.

**Implication of key findings:**

Quetiapine may be an alternative antidepressant. However, both risk and benefit of this agent should be taken into account for an individual patient with MDD.

## Background

Major depressive disorder (MDD) is a common mental illness with a lifetime prevalence rate of 6.7% (3.8% for men and 7.5% for women) [1]. As a disabling, recurrent, and chronic condition, it is a major burden for individuals, family members, communities and health care services [2,3]. In 2000, depression was an important cause of disease burden accounting for 4.4% of the total disability adjusted life years or 12% of all total years lived with disability worldwide [4].

Common classes of agents for the treatment of MDD include selective serotonin reuptake inhibitors (SSRIs) [5,6], serotonin-norepinephrine reuptake inhibitors (SNRIs) [7,8], tricyclic antidepressants (TCAs) [9-11] and monoamine oxidase inhibitors (MAOIs) [12]. Several meta-analytic findings suggest that patients with MDD may not fully respond and/or do not fully remit after receiving adequate doses and duration of these antidepressants. Only 30%-55% of MDD patients achieve remission state at the end of acute SSRI or SNRI treatment [13,14]. In addition, the overall dropout rates and the dropout rates due to adverse events are relatively high in the ranges of 25-39% and 9-17% [15,16], respectively, which suggest that many MDD patients cannot accept or tolerate currently available antidepressants [17]. While these antidepressants presumably affect serotonin and norepinephrine neurotransmitters, several lines of evidence support that dopamine neurotransmitters may also play an important role in the treatment of MDD patients [18,19].

Quetiapine and its mainly active metabolite, N-desalkylquetiapine (norquetiapine), have various pharmacological effects on central serotonergic and dopaminergic receptors, which presumably involve in its efficacy for the treatment of schizophrenia [20]. Recently, norquetiapine has been found to be a potent inhibitor for norepinephrine transporter shared commonality with TCAs and SNRIs, and a moderate-to-high affinity for D2, 5HT1A, 5HT2A, and 5HT2C receptors shared some properties with SSRIs [20,21]. These mechanisms of action may explain its efficacy for the treatment of depression and anxiety [21-24].

Several lines of research suggest that quetiapine may have an antidepressant effect. Some clinical trials found that quetiapine reduced depressive symptoms considerably in schizophrenic patients [25] and bipolar depression [26,27]. Although some experts view that its antidepressant effect may not be superior to other antidepressants [28], this is the only agent approved for the treatment of acute bipolar depression [29].

Recently, some randomized-controlled trials of quetiapine have been conducted in patients with MDD [30-33]. Since most of these studies have small samples, a meta-analysis, which is more powerful in estimating the true effect size, may be a strategy to confirm its efficacy and safety.

To determine the efficacy, acceptability and tolerability of quetiapine monotherapy in patients with MDD, we conducted a systematic review of randomized, placebo-controlled trials of quetiapine in these patients.

## Methods

The authors searched such clinical trials carried out between 1991 and February 2012.

### Eligibility criteria

The included studies were randomized, placebo-controlled trials of quetiapine monotherapy in adults (18–65 years old) with MDD. Depressive severity was rated at baseline and during treatment with standard rating scales [34] and reported the response rate, the overall discontinuation rate or the discontinuation rate due to adverse events were included. Studies with any duration of treatment for single or recurrent major depressive episodes diagnosed by any set of criteria were included. No language restriction was applied.

### Information sources

We searched MEDLINE, EMBASE, CINHL, PsycINFO and Cochrane Controlled Trials Register databases in February 2012. According to MEDLINE search, the first publication of the ICI 204,636 (the drug code of quetiapine) was in 1991. The searches, therefore, covered the period of 1991- February 2012. Searches were limited to humans and adults only. Additional studies were also searched from the databases of AstraZeneca, the producer of the original quetiapine. References of the articles obtained by any means were searched. All relevant randomized-controlled trials (RCTs) and clinical-controlled trials (CCTs) were included.

### Searches

The MEDLINE search strategies for optimal sensitivity in identifying randomized clinical trials used the following words and phrases: [(quetiapine) OR (seroquel)] AND [(major depressive disorder) OR (MDD) OR (major depression) OR (severe depressive episode)]. Similarly, these search strategies were used in the rest of databases.

### Study selection

Two reviewers (NM and BM) independently examined all abstracts identified by electronic searches to determine those meeting the eligible studies described above. The full-text articles of relevant studies were obtained. The reviewers then independently assessed all full study reports. Disputes were resolved through consensus.

### Data collection process

We developed a data extraction form. A reviewer (NM) extracted the data into the form. The second reviewer (BM) checked the extracted data. Any disputes between reviewers were resolved by consensus. If no agreement could be reached, the third author (MS) would decide.

### Data items

Extracted data obtained from each trial included: (1) study details needed for validity assessment; (2) baseline characteristics of participants, diagnostic criteria, study design and inclusion/exclusion criteria; (3) form, dose, and duration of quetiapine compared to placebo; (4) outcomes of interest. As far as possible, the intention-to treat results were recorded.

### Risk of bias in individual studies

Two reviewers (NM and BM) rated the internal validity (quality) of each eligible randomized trial. Based on the Cochrane Collaboration quality assessment for trials, risks of bias were determined by: 1) the quality of randomization, 2) concealment of allocation, 3) blinding, 4) baseline similarity, 5) intention-to-treat analysis, 6) free of selective reporting, and 7) free of other biases [35]. Studies with two risks or less were included in the analysis.

### Summary measures

Outcomes of interest included efficacy, acceptability and tolerability. Efficacy measures relied on the mean change scores of a rating scale for MDD and the response rates defined by any set of criteria. Since acceptability and tolerability have been used interchangeably, both terms were specifically defined. Similar to a previous meta-analysis, the acceptability in this review was derived from the overall discontinuation rate [17]. The tolerability, frequently defined as a measure of antidepressant side effects [36], was drawn from the discontinuation rate due to adverse events.

Relative risks (RRs), the ratios of the probability of the event occurring in the experimental (intervention) group versus the controlled group, with the 95% confidence interval (95%CI) were used for synthesizing the dichotomous data. A relative risk of one indicates no difference between comparison groups. For an unwanted outcome, an RR lesser than one suggests the less likelihood of the occurrence of such outcome. In this meta-analysis, relative risks were used to compare the response rates, overall discontinuation rates, and discontinuation rates due to adverse events between groups. In addition to the measure of efficacy, the number needed to treat (NNT), the number of patients who must receive a particular therapy for one to benefit, was also calculated.

A weighted or standardized mean difference (WMD or SMD) is the difference between two means divided by an estimate of the within group standard deviation; this mean difference, with 95%CI, was used for the synthesis of continuous data. When an outcome is measured by different instruments across studies, it may not be possible to compare or combine study results directly. By expressing the effect as a standardized value, the results can be combined since they have no unit. As a function of direct comparison or combination of trials, a WMD is plausible when the same instrument of measurement was used. In the present review, the WMDs or the SMDs were planned to be calculated if the retrieved studies used the same rating scale or different rating scales, respectively. When a standard deviation (SD) for mean change of depressive rating score was not provided, we estimated the SD by using direct substitution or any statistical method [37].

### Synthesis of results

Both fixed effect and random effect models can be used for data synthesis. For the fixed effect approach, we assume that all included studies share a common effect size. The ignorance of this between-study variation is also the main difference from a random effect model. For most instances, the assumption of one true effect size may be implausible. Although the included studies are relatively similar, there is generally no reason to assume that they are exactly the same. For this reason, a random effect model was applied for the synthesis of data in this review.

### Risk of bias across studies

To detect the reporting biases, a funnel plot, a simple plot of the intervention effect estimating from individual studies against some measure of each study’s size or precision [38], was applied.

### Test of heterogeneity

A test of heterogeneity is necessary for evaluating the similarity of the study results. Before conducting this meta-analysis, we hypothesized that the effect size may differ due to the methodological quality of the studies. We checked whether the study results had greater differences than expected by chance alone. This was done by observing the results presented by graphical display and using the test of heterogeneity. An I^2^ of 50% or higher was considered as significant heterogeneity of results.

### Statistical software

All analyses were conducted using the RevMan 5.1 (The Nordic Cochrane Centre, Copenhagen, Denmark).

## Results

### Study selection

**Figure 1:**
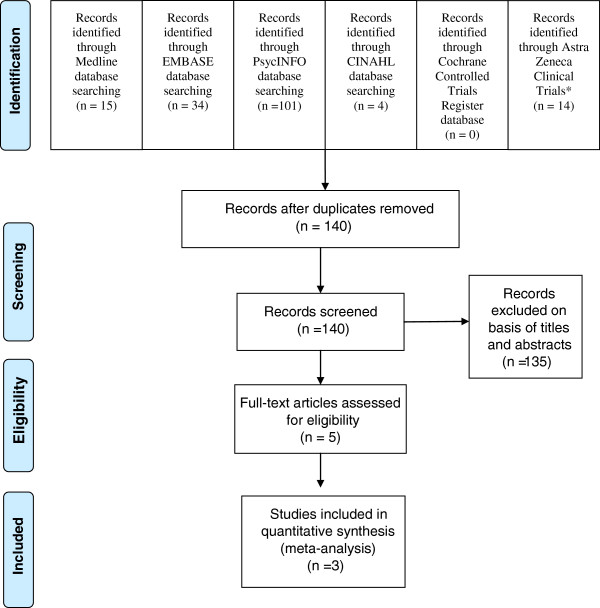
Flow diagram of study * http://www.astrazenecaclinicaltrials.com/drug-products/seroquel/.

Database searches provided a total of 168 citations (Medline = 15 studies, EMBASE = 34 studies, CINHL = 4 studies, PsycINFO = 101 studies, Cochrane Controlled Trials Register = 0 study and Astra Zeneca Clinical Trials = 14) (see Figure 1). After the remove of duplicates, 141 trials remained. By reviewing the titles and abstracts, 136 trials were excluded since they clearly did not meet the criteria. Full papers of five studies were examined [30-33,39], two studies were excluded since one was a study of maintenance treatment [30] and the other studied in geriatric patients [39], only three trials [31-33], therefore, were included in the review. No relevant, unpublished study meeting the inclusion criteria was identified.

### Study characteristics

The study periods of the three trials ranged from 8 to 11 weeks. All studies had wash-out periods (up to 4 weeks) for discontinuing psychotropic medications followed by randomization and a 2-week, post-treatment, drug-discontinuation phase.

Of 1,497 participants, 64.3% were female. All participants were outpatients with MDD, single or recurrent episode. None of them had treatment-resistant depression. Mean ages of the quetiapine and placebo groups were 41.38 (SD = 11.68) and 41.66 (SD = 11.72), respectively. The patients in all studies were treated with the extended-release (XR) form of quetiapine. The dosage of quetiapine XR ranged from 50 to 300 mg/day. Table 1 shows the main characteristics of included studies.

**Table 1:** The important characteristics of controlled trials of quetiapine in major depressive disorder

**Study (author, year)**	**Number of patients**	**Age of subjects (years)**	**Study duration (weeks)**	**Drug /Dose**	**Diagnostic criteria**	**Respond criteria**	**Remission criteria**	**Outcome measures**
Cutler AJ [2009]	461	18–65	8	Quetiapine XR/150 and 300 mg/day	DSM IV	≥ 50% reduction in MADRS	MADRS ≤ 8	MADRS, HAM-D, HAM-A, CGI-S, CGI-I, PSQI, SAS, BARS, CSFQ, 18-item TDSS scale
Weisler R [2009]	700	18–65	8	Quetiapine XR**/**50, 150 and 300 mg/day	DSM IV	≥ 50% reduction in MADRS	MADRS ≤ 8	MADRS, HAM-D, HAM-A, CGI-S, CGI-I, PSQI, SAS, BARS, CSFQ, Q-LES-Q, 18-item TDSS scale
Bortnick B [2011]	299	18–65	10	Quetiapine XR**/**150 to 300 mg/day	DSM IV	≥ 50% reduction in MADRS	MADRS ≤ 8	MADRS, HAM-D, HAM-A, CGI-S, CGI-I, PSQI, SAS, BARS, CSFQ, Q-LES-Q, 18-item TDSS scale

MADRS = Montgomery-Åsberg Depression Rating Scale; HAM-D = Hamilton Rating Scale for Depression; HAM-A = Hamilton Rating Scale for Anxiety; CGI-S= Clinical Global Impression–Severity of Illness; CGI-I = Clinical Global Impression–Improvement scale; PSQI = Pittsburgh Sleep Quality Index; SAS = Simpson-Angus Scale, BARS = Barnes Akathisia Rating Scale; CSFQ = Changes in Sexual Functioning Questionnaire; Q-LES-Q = Quality of Life Enjoyment Satisfaction Questionnaire; TDSS scale = Treatment discontinuation.

Since all three studies reported mean change scores of Montgomery-Asberg Depression Rating Scale (MARDS) and Hamilton Depression Rating Scale (HAM-D), the WMDs of mean change scores were calculated and synthesized. Remission rate, response rates, overall discontinuation rates, and discontinuation due to adverse events were also reported in all studies.

### Risk of bias within studies

All studies applied the technique of randomization, double-blind placebo control, and modified intention-to treat analysis. Dropout data and baseline similarity were reported in all studies. Only two studies [32,33] reported sequence generation of randomization and allocation concealment. No risk of bias for baseline similarity and other bias were found (see Table 2). As all studies had low risks of bias, the data of all three trials were included in the analysis.

**Table 2:** Risk of bias summary of controlled trials of quetiapine in major depressive disorder

**Study**	**Issues of bias**							
	**1**	**2**	**3**	**4**	**5**	**6**	**7**	**8**
Cutler AJ [2009]	N	N	N	N	N	N	N	N
Weisler R [2009]	N	N	N	N	N	N	N	N
Bortnick B [2011]	N/A	N/A	N	N	N	N	N	N

1=Adequate sequence generation; 2 = Allocation concealment; 3=Blinding (Subjective outcome); 4 = Dropout data addressed; 5 = Free of selective reporting; 6 = Free of other bias; 7 = Baseline similarity; 8 = Intention-to-treat analysis or modified intention-to-treat analysis; N/A = not available; N = No risk of bias.

### Results of individual studies

**Figure 2:**
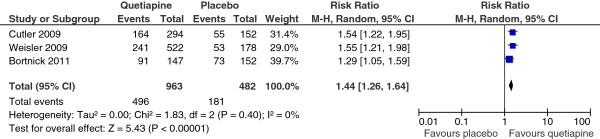
Comparison of relative risk (95% confidence interval) for clinical response rates in patients with MDD: quetiapine vs. placebo.

**Figure 3:**
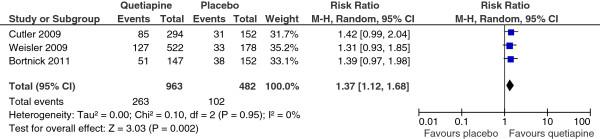
Comparison of relative risk (95% confidence interval) for clinical remission rates in patients with MDD: quetiapine vs. placebo.

**Figure 4:**
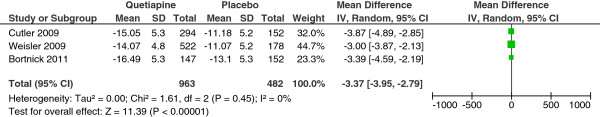
Comparison the mean changes from baseline of MADRS (95% confidence interval) in patients with MDD: quetiapine vs. placebo.

**Figure 5:**
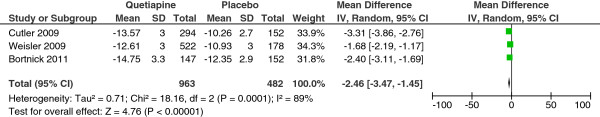
Comparison the mean changes from baseline of HAM-D (95% confidence interval) in patients with MDD: quetiapine vs. placebo.

The response rate of the quetiapine-treated group in each study was significantly greater than that of the placebo-treated group (see Figure 2). However, the remission rate of the quetiapine-treated group in each study was not significantly higher than that of the placebo-treated group (see Figure 3). The mean change MADRS and HAM-D scores of the quetiapine-treated group in each study were higher than that of the placebo-treated group (see Figures 4 and 5).

### Synthesis of results

#### Efficacy

No significant heterogeneity was found on all efficacy outcomes, except HAM-D scores. The pooled response rate of the quetiapine -treated group was significantly greater than that of the placebo-treated group [RR (95%CI) of 1.44 (1.26, 1.64), I^2^ = 0%] (see Figure 2). The pooled remission rate of the quetiapine-treated group was also significantly greater than that of the placebo-treated group [RR (95%CI) of 1.37 (1.12, 1.68), I^2^ = 0%] (see Figure 3). The pooled mean change MADRS score of the quetiapine-treated group was significantly higher than that of the placebo-treated group [WMD (95%CI) of -3.37 (-3.95, -2.79), I^2^ = 0%] (see Figure 4). The pooled mean change HAM-D score of the quetiapine-treated group was also significantly higher than that of the placebo-treated group [WMD (95%CI) of -2.46 (-3.47, -1.45), I^2^ = 89%] (see Figure 5). With respect to sleep time, the pooled mean change Pittsburgh Sleep Quality Index (PSQI) score of the quetiapine-treated group was also significantly higher than that of the placebo-treated group [WMD (95%CI) of -1.21 (-1.81, -0.61), I^2^ = 48%]. According to the pooled response rate obtained from all trials, the NNT (95%CI) was 7.2 (5.2-11.7).

#### Discontinuation rates

There was no significant heterogeneity on both discontinuation outcomes. The pooled overall discontinuation rate of the quetiapine group was not significantly different from that of the placebo group [RR (95%CI) of 1.16 (0.97, 1.39), I^2^ = 0%]. However, the pooled discontinuation rate due to adverse events in the quetiapine group was significantly higher than that of the placebo group [RR (95%CI) of 2.90 (1.87, 4.48), I^2^ = 0%].

### Risk of bias across studies

Tests for funnel plot asymmetry is normally used in the meta-analysis with at least 10 studies included; the power of the tests is too low to distinguish chance from real asymmetry, if fewer studies [38]. Because only 3 studies were included in this analysis, a test for funnel plot, therefore, was not done.

## Discussion

Our review found only three randomized, placebo-controlled trials of quetiapine carried out in adult patients with MDD. All studies had the same study designs. The preliminary findings of this meta-analysis suggest that quetiapine monotherapy is effective for adult patients with MDD. Based on the pooled response rates, the NNT of 7.2 indicates that, by average, every 1 in 8 patients with MDD will respond to quetiapine treatment. The high dropout rate due to adverse events suggests that some MDD patients may not be able to tolerate quetiapine XR. Due to the balance of its efficacy benefit and risk of side effects, as the overall discontinuation rate shown, the acceptability of this agent is not more than placebo.

In this review, the pooled response rate for quetiapine monotherapy in MDD patients (51.51%) was superior to placebo (37.55%). Similarly, a meta-analysis of 7 RCTs found that the response rates of bupropion and SSRIs treatment in MDD patients were 62 and 63%, respectively, compared with 51% for placebo [40]. The remission rate of quetiapine treatment (27.31%) is also greater than that of placebo one (21.16%). Previous meta-analyses found that the remission rates of TCAs, SSRIs, and SNRIs were 44.1, 37.7 and 31-55% respectively [41]. It should be noted that not only the response and remission rates of quetiapine treatment but also those of placebo treatment found in this study were lower than previous findings. Therefore, the cross-trial comparisons of the response and remission rates of quetiapine with those of other antidepressants may not be justified.

Absolute risk reduction (ARR) is the difference between the control group’s event rate and the experimental group’s event rate. As a measure of the size of difference between two treatments (e.g., antidepressants and placebo), the ARR may be a logical method for comparing the remission and response rates of antidepressants across studies. Based on the ARR, quetiapine increases the response rate for 13.96% (51.51% for quetiapine group vs. 37.55% for placebo group), while bupropion and SSRIs increase the response rates for 11% and 12%, respectively (62% for bupropion group and 63% for SSRI group vs. 51% for placebo group) [40]. Regarding the response rates of other antidepressants in adults with MDD, the NNTs of fluoxetine and venlafaxine are between 5 and 7, which are comparable to the NNT of 8 for the quetiapine treatment in this population [42]. Not surprisingly, as an inverse of the ARR, the comparable of ARRs between quetiapine and other antidepressants, the NNTs of these agents are, therefore, comparable. However, the ARR for the remission rate of quetiapine treatment [6.15% (27.31% for quetiapine vs. 21.16% for placebo)] is relatively lower than those of SSRIs and bupropion [11% (47% for SSRIs and bupropion vs. 36% for placebo)] [40]. It is not yet known whether these differences reflect the true lower remission rate of quetiapine treatment or only the dissimilarity of participants and study designs among the studies.

Similar to other antidepressants, the acceptability of quetiapine for the treatment of MDD is not higher than that of placebo. The nonsignificant differences in this respect have been found in a number of meta-analyses of paroxetine [43] and duloxetine [44] and TCAs [10] for the treatment of MDD. The low tolerability of quetiapine treatment for MDD is also similar to previous findings of other antidepressants, e.g. TCAs [45], SSRIs [45] and SNRIs [46,47]. These lines of evidence suggest that almost all antidepressants, including quetiapine, have the same profile of comparable acceptability to and less tolerability than placebo.

Sleep disturbance is common in MDD and may complicate its treatment. Up to 31% of MDD patients may have insomnia [48]. In addition, insomnia is a common adverse effect of many antidepressants, e.g., SSRIs [49] and SNRIs [50]. The findings of sleep quality improved by quetiapine suggests an advantage of quetiapine for the treatment of MDD.

There were several limitations of this review. Firstly, the doses of quetiapine were different across the included studies. While a study used a flexible dose with titration, the others applied fixed and multiple doses in several arms. Secondly, this meta-analysis included only three RCTs sponsored by a pharmaceutical company holding the patent of quetiapine XR. These results, therefore, should be viewed as the very preliminary findings. Thirdly, although all studies adhered to the same methods of double blindness/randomization, had similar baseline characteristics, and applied the intention-to-treat analysis or modified intention-to-treat analysis for the data analyses, 1 of 3 studies did not report an adequate sequence generation of randomization and the allocation concealment. Fourthly, as with all meta-analyses, publication bias must be considered. However, due to the small number of included studies, we did not assess the possibility of publication bias [38].

## Conclusions

Based on the limited evidence obtained from three RCTs, quetiapine XR is effective for adult patients with MDD. The high dropout rate due to adverse events suggests that some MDD patients may not be able to tolerate quetiapine XR. Due to the balance of its efficacy benefit and risk of side effects, as the overall discontinuation rate shown, the acceptability of this agent is not more than placebo. These results should be viewed as the very preliminary one. Further studies in this area are warranted.

## Competing interest

NM has received travel reimbursement from GlaxoSmithKline, Pfizer, Janssen-Cilag and Lundbeck. BM has received honoraria and/or travel reimbursement from GlaxoSmithKline, and Pfizer. MS has received honoraria, consultancy fees, research grants, and/or travel reimbursement from AstraZeneca, GlaxoSmithKline, Pfizer, Janssen-Cilag, Johnson & Johnson, Lundbeck, Thai-Otsuka, Sanofi-Aventis, and Servier. SDM has received honoraria, consultancy fees and research grants from Astra Zeneca, Eli Lilly, Janssen-Cilag, Sanofi-Aventis, Novartis and Wyeth.

## Authors’ contributions

All authors conceived the idea, prepared the study protocol, prepared the manuscript, and approved the manuscript in its current form. NM and BM searched the databases, extracted the data, and analysed the data.

## Funding

This review had no funding.

## Pre-publication history

The pre-publication history for this paper can be accessed here:

http://www.biomedcentral.com/1471-244X/12/160/prepub
